# Mediastinal parathyroid carcinoma: a case report and review of the literature

**DOI:** 10.1186/s12902-023-01363-w

**Published:** 2023-06-06

**Authors:** Yan Bao, Ganjun Kang, Xiaoyan Wu, Jing Li, Yan Huang, Ye Wang

**Affiliations:** 1 Department of Endocrinology, Renmin Hospital of Wuhan University, Wuhan, Hubei China; 2 Present address: Department of Endocrinology, Renmin Hospital of Wuhan University, Jiefang Road 238, Wuhan, Hubei 430060 China; 3 Departments of Thoracic Surgery, Renmin Hospital of Wuhan University, Wuhan, Hubei China; 4 Department of Pathology, Renmin Hospital of Wuhan University, Wuhan, Hubei China

**Keywords:** Primary hyperparathyroidism, Parathyroid carcinoma, Ectopic, Mediastinum, Case report

## Abstract

**Background:**

Parathyroid carcinoma (PC) is an uncommon cause of primary hyperparathyroidism (PHPT) and particularly rare in the mediastinum. Herein, we present a case of mediastinal PC and conduct a related literature review.

**Case presentation:**

We described a case of a 50-year-old female patient with PHPT due to mediastinal PC. She was initially admitted to a local hospital in her hometown with hypercalcemia and high blood concentrations of PTH (parathyroid hormone). The patient underwent neck parathyroidectomy and pathological examination suggested parathyroid adenoma. Although the overproduction of serum calcium and PTH declined after the surgery, calcium and PTH increased again one month later, so the patient was transferred to our hospital. A 99^m^Tc-sestamibi scan revealed an ectopic finding in the mediastinum, which was also indicated on the CT image. After removing the mediastinal mass, the metabolism of calcium and PTH quickly reverted to normal and the pathologic features of the mass were consistent with PC. By reviewing the related literature, we noticed that only scattered reports were published before 1982, and those were not included in the present review due to their differences with current radiological examination and treatment methods. After excluding outdated studies, we summarized and analyzed 20 reports of isolated mediastinal PC and concluded that. Parathyroidectomy remains the only curative treatment for the disease. Furthermore, the success of treatment directly depends on accurate preoperative localization.

**Conclusion:**

With this study, we emphasize the importance of accurate preoperative diagnosis of mediastinal PC and improve clinicians’ understanding of the disease.

## Background

Parathyroid carcinoma (PC) is a rare endocrine disease, that accounts for 1-5% of all causes of sporadic primary hyperparathyroidism (PHPT) in North America, major western countries, and in Japan [1, 2]. Only 6–16% of parathyroid tumor can be found in ectopic locations such as the thyroid, the thymus, or behind the esophagus [3], and the condition rarely affects the mediastinum [4].

PC generally develops slowly [5], and many tumors are hormonally functional, which means they can induce excessive synthesis and secretion of parathyroid hormone (PTH) and hyper calcium, affecting multiple systems and organs. Patients may manifest a variety of symptoms, including gastrointestinal discomfort, osteoporosis, bone pain and pathologic fracture [6]. The diagnosis of mediastinal PC is typically very challenging, because of the nature of the disease (slow growth, and atypical symptoms in some patients) and ectopic locations for the parathyroid glands [7].

In this study, we reported an unusual case of PHPT caused by PC in the mediastinum and reviewed the related literature to raise awareness of disease diagnosis.

## Case presentation

A 50-year-old female was initially admitted to a local hospital in her hometown with a 3-month history of nausea, emesis, asthenia and moderate backache. High levels of serum calcium (4.3 mmol/L, with the normal range at 2-2.8 mmol/L) and PTH (129.5 pmol/L, normal, 1.6–6.9 pmol/L) were detected. Although neck ultrasonography revealed an increase in the volume of the parathyroid glands, no further examinations were performed at the time to determine whether there was a possibility of ectopic lesions. A left-sided parathyroidectomy and bilateral subtotal thyroidectomy were subsequently performed and pathological examination revealed parathyroid adenoma (2 × 1.5 cm) and nodular goiter. The patient’s hypercalcemia was resolved with decreasing levels of PTH observed after the operation. However, the woman developed more sever nausea and asthenia one month later, and her levels of serum calcium and PTH increased again.

After the patient was referred to our hospital, our priority was to lower her serum calcium levels while initiating diagnostic procedures to elucidate the lesion site as soon as the hypercalcemic crisis had been diagnosed. Physical examination showed that the patient’s vital signs were normal but reveled that she had difficulties in walking. However, no lumps were identified on her neck. Laboratory assessments revealed a serum calcium level of 4.63 mmol/L (normal, 2.11–2.52 mmol/L), urinary calcium level of 15.7 mmol/24 h (normal, 2.5–7.5 mmol/24 h), serum PTH of 1562.3 pg/mL (normal, 8.5–88 pg/mL), serum BUN of 13.9 mmol/L (normal, 2.6–7.5 mmol/L), and serum creatinine of 174 umol/L (normal, 41–73 umol/L).

**Fig. 1:**
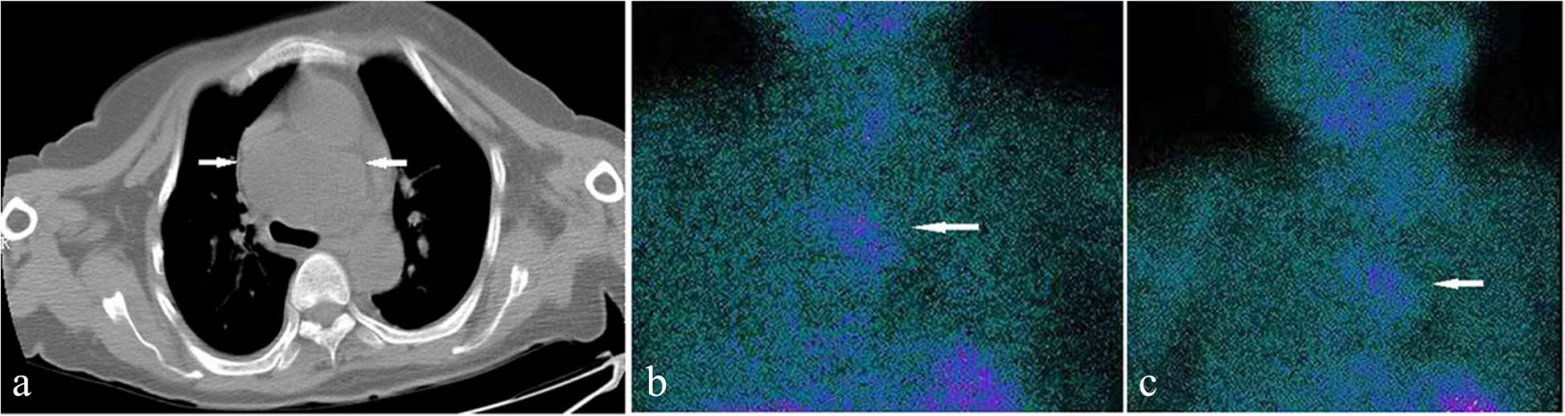
CT imaging and scintigraphic analysis using technetium-99 m. **a **A mass is found in the mediastinum on CT imaging. **b**, **c** Parathyroid scintigraphy shows high focal radiotracer uptake at 15 and 90 min  after injection of 99mTc-sestamibi

**Fig. 2:**
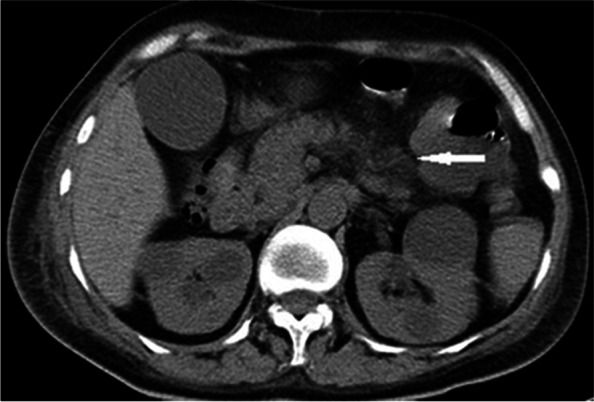
CT image of the abdomen. The CT scan image shows an enlarged pancreas with indistinct boundaries and surrounding exudates

X-ray revealed a previously undetected fracture of the left superior pubic ramus, and examination of bone mineral density (BMD) using dual-energy X-ray absorptiometry revealed decreased density in the lumbar spine (T score = − 2.6). Furthermore, ultrasonography of the patient’s urinary system revealed the presence of bilateral renal calculi. Although cervical ultrasonography did not detect any abnormalities, computed tomography (CT) of the chest showed a low-density mass in the upper-middle mediastinum, approximately 6.7 cm×4.8 cm in size (Fig. 1a), importantly, 99^m^Tc-hexakis-2-methoxyisobuthylisonitrile (99^m^Tc-MIBI) scintigraphy showed increased uptake in a location identical to that in the CT scan image (Fig. 1b, c). To exclude the diagnosis of multiple endocrine neoplasia (MEN), pituitary magnetic resonance imaging (MRI) and abdominal CT were performed. The images of pituitary and adrenal glands were normal, but abdominal CT revealed a sign of acute pancreatitis (Fig. 2). However, serum amylase and lipase levels were normal, and no family member carried a medical history of hyperparathyroidism, pituitary tumor, thyroid medullary carcinoma, or pheochromocytoma.

**Fig. 3:**
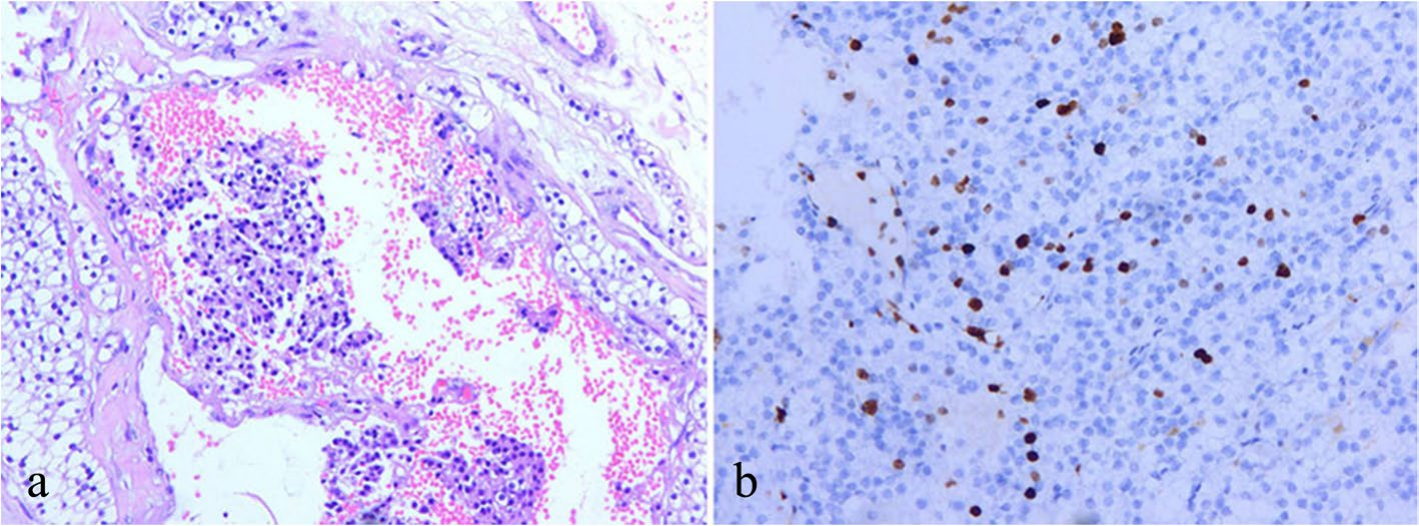
Histopathological and immunohistochemical findings. **a** Histological evaluation shows vascular invasion by parathyroid tumor cells (H&E, ×40). **b** Immunohistochemical findings show that approximately 10% of the tumor cells were Ki-67 positive (×100)

Subsequently, the patient underwent right thoracotomy and a mass measuring 6.8 cm×4.9 cm×5.3 cm was resected. The lymph node dissection was not performed since there were no obviously swollen lymph nodes seen around the tumor. Levels of serum calcium (2.42 mmol/L) and PTH (19.2 pg/mL) returned to normal after the operation. Over subsequent days, blood calcium concentrations declined, and intravenous infusion of calcium gluconate and oral vitamin D3 was administrated to correct these abnormalities. Postoperative pathological evaluation of the resected specimen disclosed a parathyroid adenocarcinoma due to vascular invasion and Ki-67 positivity in approximately 10% of the tumor cells (Fig. 3). Other immunochemical examinations showed PAX-8 (+), PTH (+), galectin-3 (-), syn (-),CgA (+), cyclin D1(+), and CEA (−).After 20 days of hospitalization, the patient was discharged and treated with oral calcium carbonate (3 g/day) and calcitriol (0.5 ug/day). She did not exhibit any signs of recurrence during a six-month follow-up.

## Literature review

We systematically searched PubMed for reports of isolated mediastinal parathyroid carcinoma in humans using the terms “parathyroid carcinoma (or cancer), mediastinal”. The cut-off time for retrieval was December 2020, and we only considered results in English language only. Furthermore, we excluded irrelevant documents (such as those on parathyroid adenoma, parathyroid hyperplasia, PC caused by secondary hyperparathyroidism) by manual screening. The first case of mediastinal parathyroid carcinoma was reported by Weissman in 1957 [8] and we identified that there were only scattered reports published before 1982, which were not included in this review due to their inherent differences with current radiological examination and treatment methods [9, 10]. Table 1 summarized 20 cases included in our final sample [11–30].

**Table 1:** Literature review of published mediastinal parathyroid carcinoma cases

Case	Age/sex	presenting symptoms	Calcium (mmol/L)	PTH (pg/ml)	size of tumor (cm)	radiological studies	surgical approach/ operation num	References
1	34/F	kidney stones, osteitis fibrosa cystica, recurrent acute pancreatitis	4.7	3304	6 × 3.5 × 3	US,CT,MIBI	transcervical surgery and median sternotomy**/**3	Jiajue R et al [11]
2	53/F	backache, multiple fractures, nausea, vomiting, fatigue, and unexplained myocardial ischemia-like symptoms.	3.7	>1900	4 × 3 × 2.5	US, MRI,MIBI	sternotomy**/**1	Xin Y et al [12]
3	53/M	fatigue, polyuria, night sweats and renal stones	3.4	2630	5 × 3 × 3	US,CT,MIBI	transthorascopic surgery/1	Cao C et al [13]
4	72**/**M	severe epigastric pain	3.5	168	2 × 1.5 × 1	US, MRI,MIBI	lower cervical collar incision/1	Tseng CW et al [14]
5	54**/**M	fatigue, ostealgia and myalgia	6.2	**/**	4 cm in diameter	**/**	NA	Peshev ZV et al [15]
6	28**/**M	nausea, ostealgia, fatigue, edema	5.83	2307	3.7 × 3.2 × 4.3	CT, MIBI	thoracotomy**/**1	Meng Z et al [16]
7	23**/**M	recurrent fractures and osteoporosis	3.58	>2306	3.6 × 2.5	US, CT,MIBI	median sternotomy/1	Yong T et al [17]
8	61**/**M	kidney stones	1.9	1220	0.85 × 0.5 × 0.38	CT, MIBI	neck collar incision/1	Iwata T et al [18]
9	10**/**boy	/	>3.75	>3000	2 × 2 × 2.5	US, MIBI	exploration of the neck**/**1	Righi A et al [19]
10	66/F	bone and joint pain	2.83	124	3 cm in diameter	US,CT,MIBI	median sternotomy/1	Damadi A et al [20]
11	84**/**F	Progressive dyspnea, severe osteoporosis with hip and vertebral fractures	2.8	230	**/**	CT,MR angiography	NA	Vazquez FJ et al [21]
12	55**/**M	fatigue, weight loss, mild lumbar pain, severe uremia	2.49	2807	2.7 cm in diameter	CT, MIBI	no detailed information/2	Tkaczyk M et al [22]
13	27**/**M	skeletal symptoms	2.96	1412	**/**	CT, MIBI	exploration of the neck and median sternotomy**/**3	Srouji IA et al [23]
14	33**/**M	fatigue, decreased motivaton	3.35	722	3 × 2 × 1	CT, MIBI	exploration of the neck **/**1	Chandran M et al [24]
15	43**/**M	thirst, polyuria	3.53	**/**		MIBI	no detailed information/2	Yamashita K et al. [25]
16	44**/**M	hematuria, flank pain	4.14	964.81	5 × 5 × 2.5	US, MIBI	exploration of the neck and median sternotomy**/**2	Delaney SE et al [26]
17	62**/**F	recurrent renal colic, thirst, vague abdominal pain, hoarseness	3.62	275	3 cm in diameter	CT	exploration of the neck and median sternotomy**/**2	Kelly MD et al [27]
18	47**/**M	shortness of breath, hoarseness, dysphagia	4.38	6500	**/**	CT	exploration of the neck and median sternotomy**/**1	Putnam JB et al [28]
19	57/F	marked osteopenia, fatigue	4.06	>3000	5 cm in diameter	CT	exploration of the neck and sternotomy**/**2	Kastan DJ et al [29]
20	51**/**M	hoarseness, productive cough, dyspnea on exertion	**/**	**/**	a large mass (no exact value)	chest x-ray	no detailed information/1	Murphy MN et al [30]

## Discussion and conclusions

In the majority of patients, PHPT is caused by benign adenomas that are located close to the thyroid gland [31]. Approximately 6-30% of patients with PHPT have ectopic parathyroid glands [32], but when it comes to parathyroid cancer, a slow-growing malignancy, accounts for less than 1% of the causes of hyperparathyroidism [33]. Ectopic parathyroid glands are found in some anatomic locations of the body, such as the mediastinum, and intrathymic and retro/paraesophageal sites [34], which can be explained by their embryologic origin [34, 35]. The parathyroid glands originate from the endodermal tissue and develop in association with the thymus from the third pharyngeal pouch, and may then migrate into the mediastinum [36]. Cases of mediastinal PCs are rare, which makes them difficult to diagnose. They are usually sporadic, while the familial form is observed in approximately 5% of cases. Among the reports we accessed for our literature review, only one reported a case of familial form in an individual diagnosed with FIHP (familial isolated hyperparathyroidism) [25].

The clinical manifestations of parathyroid carcinoma are majorly caused by the effects of excessive secretion of PTH from the tumor rather than by the infiltration of the tumor into other organs [16]. Thus, signs and symptoms of hypercalcemia are often reported, which include renal stone formation, and gastric /duodenal ulceration commonly accompanied by digestive discomfort, fatigue, weight loss, backache, osteoporosis, and fracture [6], as we noticed in our patient. High levels of serum calcium and parathyroid hormone are the most recognizable and characteristic abnormalities of the biochemical features of PCs. Compared with patients with adenomatous PHPT, patients with parathyroid carcinoma usually exhibit much higher serum calcium levels, which may then lead to hypercalcemic crisis [37, 38]. The proportion of cases with a serum calcium level greater than 3.5 mmol/L was as high as 60% (12/20) in our reviewed cases (Table 1). However, cases of non-secretory parathyroid cancer in the mediastinum also have to be considered [30] and that is why a diagnosis of PC cannot rely solely on the clinical manifestations and related biochemical abnormalities, and shall also involve the results of pathological examination after performing the resection of the gland.

Currently, surgical interventions is the best treatment for PHPT caused by mediastinal parathyroid carcinoma and the success of the procedure largely depend on the precise localization of the parathyroid tumor. Therefore, accurate preoperative diagnosis of ectopic lesions is critical part of the recovery process. Localization examinations include ultrasonography, CT, MRI and parathyroid scintigraphy with 99^m^Tc-MIBI, the latter being the most effective method, especially with ectopic parathyroid lesions [39]. The sensitivity rate of 99^m^Tc-MIBI is 68-86% and its specificity rate reaches as high as 98.3% in localization of parathyroid adenomas [40, 41]. False positives may appear in thymomas, which are usually rich in mitochondria that absorb the tracer well [42], whereas negative results are more common in multiple adenomas or hyperplasia [43]. Our literature review revealed that 14 patients were performed with 99^m^Tc-MIBI, and the results were all positive. However, the effectiveness of this technique depends on the size of the gland. It is worth mentioning that four-dimensional CT and 3T MRI can be favorable supplemental techniques to improve this process [40, 44].

Our patient demonstrated relevant clinical manifestations, and the markedly elevated PTH levels and hypercalcemia led to the preoperative suspicion of PC. Unfortunately, the local surgeons did not properly investigate the location of the tumor and then directly removed the parathyroid gland in the neck. At our department, a mass in the mediastinum identified with CT scan showed an elevated uptake rate of 99mTc-MIBI, which eventually confirmed the diagnosis of parathyroid carcinoma. If hyperparathyroidism is diagnosed, clinicians should consider the existence of ectopic glands and improve relevant examinations to avoid unnecessary harm to the patient.

The most effective therapy is undoubtedly complete surgical resection, which is recommended for all symptomatic patients and most asymptomatic patients [6, 40, 42], and a variety of surgical approaches to remove tumors have already been explored. Traditionally, median sternotomy or thoracotomy involving an open operation was used for patients with deep mediastinal parathyroid lesions. Recently, thoracoscopic surgery was considered a safe and feasible method for the resection of mediastinal parathyroid tumor [39], and a cervical approach may be appropriate for the excision of lesions located in the aortic arch or upper region [39]. Our patient underwent a traditional thoracotomy because the tumor was large and compressed the surrounding blood vessel (the superior vena cava). Intriguingly, we uncovered in our literature review of mediastinal parathyroid carcinoma that among patients with distinct records of surgical procedures, 10 out of 15 cases had undergone open chest surgery (including sternotomy or thoracotomy), which might be related to large tumor size. Furthermore, ectopic mediastinal PC is considered an important indicator associated with surgical recidivism. In the reports we analyzed, 38.9% of diagnosed cases with surgical records underwent two or more operations (Table 1). The treatments of metastatic and non-resectable PC are still limited and therapeutic methods such as chemotherapy, radiotherapy, and radiofrequency ablation of metastasis were associated with positive outcomes in several case reports [45]. When the cancer is widely metastasized and there is no opportunity for surgery, controlling hypercalcemia remains the primary treatment for patients [46].

Ultimately, the effective diagnosis of PC requires pathological examination for further confirmation, which often poses diagnostic challenges [47]. The main difficulty in histological examinations is to distinguish PC from atypical adenomas, in the latter tumors share some histological features with PC, e.g., diffuse growth pattern, fibrous septa, and high mitotic activity [45]. The unequivocal histological criteria for PC, however, should be restricted to those tumors that invade adjacent tissues, blood vessels, and perineural spaces or to those tumors that produce distant metastases [48]. In addition, immunohistochemical staining for parafibromin can help avoid diagnostic errors. The negative staining of parafibromin and overexpression of Ki-67(> 5%) are highly relevant to PC [28, 33]. In the case of our patient, her diagnosis was confirmed due to the vascular invasion and high levels of immunohistochemical Ki-67(10%).

It is equally important to acknowledge the limitations of our study. Firstly, we were unable to adequately obtain the medical records from the patient’s initial visit to her local hospital. Secondly, the patient’s diagnosis of osteoporosis was only based on the T-score of the lumbar spine, we should improve it by giving patients multi-site DXA scan (lumbar spine, femoral neck, forearm and total hip) to assess their BMD more completely. Thirdly, parafibromin immunohistochemical staining is important in the diagnosis of PC, the lack of which in our case might make the diagnosis less persuasive. Nevertheless, we consider our diagnosis is unequivocal based on the combined evidence (vascular invasion and overexpression of Ki-67). Regarding our literature review, we may still have missed a small number of cases even after conducting a careful manual screening.

Our study highlights the necessity of accurate diagnosis in the management of PC in the mediastinum by reporting an unusual case and conducting a corresponding literature review. In challenging cases like ours, ectopic parathyroid lesions (including mediastinal tumor) must be considered to avoid the need of reoperation and accompanying related risks to patients.

## Data Availability

All the data supporting our findings are contained within the manuscript.

## Abbreviations

PTCA: Parathyroid carcinoma
PHPT: Primary hyperparathyroidism
PTH: Parathyroid hormone
MEN: Multiple endocrine neoplasia
FIHP: Familial isolated hyperparathyroidism
^99m^Tc-MIBI: ^99m^Tc-hexakis2-methoxyisobuthylisonitrile
